# Supplementary material for: Genes Encoding Teleost Orthologs of Human Haploinsufficient and Monoallelically Expressed Genes Remain in Duplicate More Frequently Than the Whole Genome
Source: Int J Genomics. 2021 Jul 29;2021:9028667. doi: 10.1155/2021/9028667 (PMC8346308; doi:10.1155/2021/9028667)
Supplement: Supplementary Materials — Tables of statistic tests for each species of teleost and for each category. Each category is represented in each of the four Suppl. Data (HI, MA, X chromosome, and parental imprinting), and these four tables have the same construction. By column (category HI for example): (A) species; (B) total number of teleost genes returned in singleton; (C) total number of teleost genes remained in duplicate; (D) total number of teleost genes in triplicate or more copies; (E) total number of teleost genes with a human ortholog; (F) number of teleost orthologs of HI human genes returned in singleton; (G) number of teleost orthologs of HI human genes remained in duplicate; (G) number of teleost orthologs of HI human genes in triplicate or more copies; (I) total number of teleost orthologs to human HI gene; (J) chi2 value of the repartition of HI orthologs in singleton, in duplicate or more copies in comparison with the whole genome; (K) p value of chi2 test; (L) chi2 false discovery rate (FDR) by Benjamini Hochberg (BH) procedure. (M) p value of hypergeometric test between singleton and duplicate/more copies; (N) hypergeometric FDR by BH procedure; (O) p value of hypergeometric test between triplicate or more copies and duplicate or less copies; (P) hypergeometric FDR by BH procedure. The same organization of columns is used for the other categories (MA, X chromosome, and imprinted genes). Concerning for HI and MA categories, the chi2 test is significant for 55/57 and 47/57 species, respectively, and the hypergeometric test is significant for 57/57 and 50/57 species, respectively; i.e., these orthologs remain more frequently in duplicate than the whole genome. For comparison between triplicate (or more copies) and duplicate (or less copies), the hypergeometric test is significant for 5/57 (salmonids and carp) and 3/57 species, respectively (salmonid and carp as well). [file 9028667.f1.zip › 9028667.f1/9028667.f1.pdf]

Haploinsufficient

	Genome			
Species	Number of single copy genes	Number of duplicate copy genes	Number of triplicate or more copy genes	Total number of genes
Amazon molly	10313	2909	712	13934
Atlantic herring	10198	2818	624	13640
Atlantic salmon	3919	6418	4147	14484
Ballan wrasse	9759	3130	1027	13916
Barramundi perch	10123	3089	836	14048
Blue tilapia	10480	2910	651	14041
Blunt-snouted clingfish	10139	2552	549	13240
Brown trout	3744	7066	3816	14626
Burton's mouthbrooder	10678	2836	514	14028
Channel bull blenny	10109	2678	517	13304
Channel catfish	10868	2672	590	14130
Climbing perch	10638	2979	540	14157
Cod	10411	2323	379	13113
Common carp	3530	5699	4772	14001
Denticle herring	9916	3182	798	13896
Eastern happy	10464	2865	713	14042
Electric eel	10526	2973	513	14012
European seabass	10503	2861	442	13806
Fugu	10133	2549	576	13258
Gilthead seabream	10711	2870	495	14076
Greater amberjack	10750	2957	552	14259
Guppy	10623	2662	509	13794
Huchen	3797	6234	4510	14541
Indian glassy fish	10440	2833	556	13829
Indian medaka	10589	2798	531	13918
Japanese medaka HdrR	10699	2557	474	13730
Japanese medaka HNI	10528	2432	426	13386
Japanese medaka HSOK	10609	2563	455	13627
Jewelled blenny	9907	2882	881	13670
Large yellow croaker	10645	2916	464	14025
Live sharksucker	10656	2761	422	13839
Lyretail cichlid	10331	2816	640	13787
Makobe Island cichlid	10552	2881	537	13970
Mexican tetra	10464	3139	621	14224
Midas cichlid	10441	2761	595	13797
Mummichog	10489	2813	553	13855
Nile tilapia	10588	2803	574	13965
Northern pike	10447	3354	462	14263
Orbiculate cardinalfish	10081	2994	797	13872
Pachon cavefish	10333	2844	497	13674
Pinecone soldierfish	10486	3042	553	14081
Rainbow trout	4274	6304	3445	14023
Red-bellied piranha	10767	3201	589	14557
Sailfin molly	10238	2843	780	13861
Sheepshead minnow	10388	2800	597	13785

Shortfin molly	10174	2962	780	13916
Siamese fighting fish	10663	2637	432	13732
Stickleback	10384	2474	337	13195
Swamp eel	10771	2672	512	13955
Tetraodon	9699	2576	643	12918
Tiger tail seahorse	10485	2519	512	13516
Tongue sole	10473	2615	555	13643
Turbot	10755	2757	340	13852
Yellowtail amberjack	10347	3084	735	14166
Zebra mbuna	10262	3037	831	14130
Zebrafish	10601	2942	552	14095
Zig-zag eel	10768	2867	431	14066
**MAXIMAL**	10868	7066	4772	14626
**MINIMAL**	3530	2323	337	12918
**MEAN**	9853,78947	3135,28070	892,82456	13881,89474

Haploinsufficient genes			
Number of haploinsufficient teleost orthologs returned in singleton	Number of haploinsufficient teleost orthologs remained in duplicate	Number of haploinsufficient teleost orthologs remained in triplicate or more	Total number of haploinsufficient genes
181	84	11	276
175	76	9	260
50	112	119	281
179	79	18	276
170	94	11	275
193	78	6	277
185	71	7	263
48	120	111	279
187	82	11	280
189	71	11	271
183	94	4	281
190	83	7	280
191	75	5	271
47	103	124	274
169	90	15	274
186	78	13	277
168	100	7	275
186	82	5	273
183	68	7	258
188	86	3	277
184	87	9	280
190	73	14	277
49	99	140	288
181	82	10	273
182	87	8	277
194	82	6	282
193	76	7	276
189	75	6	270
180	78	12	270
190	82	3	275
188	82	3	273
177	84	12	273
186	81	10	277
169	101	8	278
182	83	13	278
190	78	8	276
187	82	6	275
168	102	6	276
180	79	15	274
173	90	5	268
182	88	11	281
56	122	98	276
176	98	9	283
179	83	15	277
193	73	13	279

Haploinsufficient

176	86	18	280
199	76	4	279
194	74	5	273
192	77	7	276
168	83	16	267
185	74	12	271
190	74	13	277
190	82	6	278
180	89	11	280
182	85	14	281
177	95	7	279
192	83	4	279
199	122	140	288
47	68	3	258
171,77193	85,10526	18,73684	275,61404

Comparison between singleton, duplicate, triplicate and more copies

Chi2 test	Chi2 test p-value	Chi2 test FDR (Benjamini-Hochberg procedure)	Hypergeometric test p-value	Hypergeometric test FDR (Benjamini-Hochberg procedure)
10,2061590470	0,0013997239	0,0022845641	0,0010470035	0,0017051200
7,6645462790	0,0056316421	0,0069783391	0,0039069775	0,0048412548
12,2186004916	0,0004731541	0,0009988809	0,0001607143	0,0005348866
3,6630379059	0,0556321099	0,0566255404	0,0323212520	0,0328984172
14,3276075756	0,0001535957	0,0006734582	0,0001301982	0,0005300925
3,6050649087	0,0576038201	0,0576038201	0,0340654876	0,0340654876
5,7028904467	0,0169369985	0,0182152625	0,0110201051	0,0118518112
10,3215136816	0,0013148840	0,0022845641	0,0004935431	0,0010047127
13,4184281030	0,0002491644	0,0007101185	0,0002242059	0,0005877859
5,7881135182	0,0161348946	0,0180331175	0,0104126534	0,0115932359
21,9973707902	0,0000027302	0,0000485706	0,0000040418	0,0000592545
7,9576927880	0,0047883414	0,0061704021	0,0033217325	0,0042501311
13,1649065383	0,0002852410	0,0007742256	0,0002798500	0,0006935414
9,4382112212	0,0021251066	0,0031059251	0,0008364602	0,0015380074
12,5616371816	0,0003937466	0,0009816221	0,0003068178	0,0007286922
7,9265831915	0,0048713701	0,0061704021	0,0033553666	0,0042501311
28,9656492186	0,0000000737	0,0000041994	0,0000001499	0,0000085421
9,4651545368	0,0020941179	0,0031059251	0,0015778364	0,0023667546
4,3308168797	0,0374282791	0,0387893075	0,0231318459	0,0239730039
10,2989277339	0,0013310753	0,0022845641	0,0010384652	0,0017051200
14,1318324627	0,0001704351	0,0006939143	0,0001559338	0,0005348866
11,0919685290	0,0008670240	0,0017650131	0,0007137438	0,0013757149
12,3569821168	0,0004393403	0,0009988809	0,0001443312	0,0005348866
12,4710189651	0,0004133146	0,0009816221	0,0003529652	0,0007941895
16,3922533403	0,0000514953	0,0003261366	0,0000540109	0,0003420691
13,6645611921	0,0002185400	0,0007101185	0,0002082674	0,0005877859
12,5030152191	0,0004062958	0,0009816221	0,0003736318	0,0007941895
9,6564791569	0,0018868490	0,0029875110	0,0014851626	0,0022879531
4,5620880621	0,0326871982	0,0345031536	0,0197480219	0,0208451343
6,9707108930	0,0082854574	0,0098389807	0,0055990507	0,0066488727
10,2021074580	0,0014028025	0,0022845641	0,0011125740	0,0017615755
14,8195401325	0,0001183033	0,0005619409	0,0001111366	0,0004872912
10,5390135780	0,0011688078	0,0022207349	0,0009110236	0,0016227608
23,3284707233	0,0000013656	0,0000389206	0,0000018364	0,0000523387
15,7381000785	0,0000727441	0,0004146414	0,0000725153	0,0004133371
7,0720880617	0,0078293959	0,0094952248	0,0052907387	0,0064164277
9,1679847138	0,0024628563	0,0034239709	0,0018215800	0,0025324405
21,5716876066	0,0000034085	0,0000485706	0,0000041582	0,0000592545
6,7181613163	0,0095435968	0,0111017350	0,0061758149	0,0071841112
17,6098233614	0,0000271184	0,0001932186	0,0000302155	0,0002152855
13,9075037655	0,0001920303	0,0007084321	0,0001689116	0,0005348866
13,5216342157	0,0002358290	0,0007101185	0,0000799658	0,0004143685
20,3711268612	0,0000063785	0,0000519392	0,0000074933	0,0000610167
12,2523989834	0,0004646604	0,0009988809	0,0003761950	0,0007941895
5,7411434700	0,0165720004	0,0181654619	0,0105762853	0,0115932359

14,9723067477	0,0001091006	0,0005619409	0,0000967950	0,0004597765
6,4307050341	0,0112164047	0,0127867014	0,0075230389	0,0085762644
9,4903784786	0,0020655221	0,0031059251	0,0016270635	0,0023780158
9,0967994609	0,0025605717	0,0034750615	0,0019291005	0,0026180649
21,1016440984	0,0000043555	0,0000496532	0,0000056708	0,0000610167
13,4996159692	0,0002386123	0,0007101185	0,0002268647	0,0005877859
10,3726061715	0,0012789887	0,0022845641	0,0010109007	0,0017051200
13,8418423699	0,0001988581	0,0007084321	0,0001912887	0,0005738661
10,9002737974	0,0009615005	0,0018898458	0,0007240605	0,0013757149
8,7252644021	0,0031383056	0,0041600795	0,0021639893	0,0028685439
20,7316023375	0,0000052837	0,0000501949	0,0000067116	0,0000610167
9,3027989029	0,0022880403	0,0032604575	0,0017167394	0,0024463536
28,96565	0,05760	0,05760	0,03407	0,03407
3,60506491	0,00000007	0,00000420	0,00000015	0,00000854
11,89211	0,00559	0,00637	0,00352	0,00407

Comparison between tripli or more copies and dupli or less copies	
Hypergeometric test p-value	Hypergeometric test FDR (Benjamini-Hochberg procedure)
0,8405420446	0,9977290843
0,8465903628	0,9977290843
0,0000004878	0,0000092687
0,7419100984	0,9977290843
0,9416467713	0,9977290843
0,9900214941	0,9977290843
0,9238422923	0,9977290843
0,0000003194	0,0000091022
0,4502664310	0,9977290843
0,4850482528	0,9977290843
0,9977290843	0,9977290843
0,9136706806	0,9977290843
0,8962819500	0,9977290843
0,0000713147	0,0008129874
0,6140214247	0,9977290843
0,6551854774	0,9977290843
0,8811426208	0,9977290843
0,9403476032	0,9977290843
0,9356512805	0,9977290843
0,9971380494	0,9977290843
0,7612433867	0,9977290843
0,1458988969	0,9977290843
0,0000000002	0,0000000132
0,6634619857	0,9977290843
0,8345244581	0,9977290843
0,9278126172	0,9977290843
0,7799939293	0,9977290843
0,8916808704	0,9977290843
0,9368565868	0,9977290843
0,9950670394	0,9977290843
0,9906273482	0,9977290843
0,6188397823	0,9977290843
0,6256966388	0,9977290843
0,9229924282	0,9977290843
0,4226425927	0,9977290843
0,8656110427	0,9977290843
0,9722945232	0,9977290843
0,8869896982	0,9977290843
0,6147665639	0,9977290843
0,9689223674	0,9977290843
0,5480569810	0,9977290843
0,0000267848	0,0003816837
0,8137519881	0,9977290843
0,5992324073	0,9977290843
0,4337543415	0,9977290843

0,3075823245	0,9977290843
0,9777122354	0,9977290843
0,8308546122	0,9977290843
0,8846251972	0,9977290843
0,2567840545	0,9977290843
0,3312017859	0,9977290843
0,3383605328	0,9977290843
0,6817055866	0,9977290843
0,8659999059	0,9977290843
0,7765715194	0,9977290843
0,9244591043	0,9977290843
0,9736627946	0,9977290843
0,99773	0,99773
0,00000000	0,00000001
0,69160	0,91023
