# Supplementary material for: Genes Encoding Teleost Orthologs of Human Haploinsufficient and Monoallelically Expressed Genes Remain in Duplicate More Frequently Than the Whole Genome
Source: Int J Genomics. 2021 Jul 29;2021:9028667. doi: 10.1155/2021/9028667 (PMC8346308; doi:10.1155/2021/9028667)
Supplement: Supplementary Materials — Tables of statistic tests for each species of teleost and for each category. Each category is represented in each of the four Suppl. Data (HI, MA, X chromosome, and parental imprinting), and these four tables have the same construction. By column (category HI for example): (A) species; (B) total number of teleost genes returned in singleton; (C) total number of teleost genes remained in duplicate; (D) total number of teleost genes in triplicate or more copies; (E) total number of teleost genes with a human ortholog; (F) number of teleost orthologs of HI human genes returned in singleton; (G) number of teleost orthologs of HI human genes remained in duplicate; (G) number of teleost orthologs of HI human genes in triplicate or more copies; (I) total number of teleost orthologs to human HI gene; (J) chi2 value of the repartition of HI orthologs in singleton, in duplicate or more copies in comparison with the whole genome; (K) p value of chi2 test; (L) chi2 false discovery rate (FDR) by Benjamini Hochberg (BH) procedure. (M) p value of hypergeometric test between singleton and duplicate/more copies; (N) hypergeometric FDR by BH procedure; (O) p value of hypergeometric test between triplicate or more copies and duplicate or less copies; (P) hypergeometric FDR by BH procedure. The same organization of columns is used for the other categories (MA, X chromosome, and imprinted genes). Concerning for HI and MA categories, the chi2 test is significant for 55/57 and 47/57 species, respectively, and the hypergeometric test is significant for 57/57 and 50/57 species, respectively; i.e., these orthologs remain more frequently in duplicate than the whole genome. For comparison between triplicate (or more copies) and duplicate (or less copies), the hypergeometric test is significant for 5/57 (salmonids and carp) and 3/57 species, respectively (salmonid and carp as well). [file 9028667.f1.zip › 9028667.f1/9028667.f2.pdf]

| | Genome | | | |
|---|---|---|---|---|
| **Species** | **Number of single copy genes** | **Number of duplicate copy genes** | **Number of triplicate or more copy genes** | **Total number of genes** |
| Amazon molly | 10313 | 2909 | 712 | 13934 |
| Atlantic herring | 10198 | 2818 | 624 | 13640 |
| Atlantic salmon | 3919 | 6418 | 4147 | 14484 |
| Ballan wrasse | 9759 | 3130 | 1027 | 13916 |
| Barramundi perch | 10123 | 3089 | 836 | 14048 |
| Blue tilapia | 10480 | 2910 | 651 | 14041 |
| Blunt-snouted clingfish | 10139 | 2552 | 549 | 13240 |
| Brown trout | 3744 | 7066 | 3816 | 14626 |
| Burton's mouthbrooder | 10678 | 2836 | 514 | 14028 |
| Channel bull blenny | 10109 | 2678 | 517 | 13304 |
| Channel catfish | 10868 | 2672 | 590 | 14130 |
| Climbing perch | 10638 | 2979 | 540 | 14157 |
| Cod | 10411 | 2323 | 379 | 13113 |
| Common carp | 3530 | 5699 | 4772 | 14001 |
| Denticle herring | 9916 | 3182 | 798 | 13896 |
| Eastern happy | 10464 | 2865 | 713 | 14042 |
| Electric eel | 10526 | 2973 | 513 | 14012 |
| European seabass | 10503 | 2861 | 442 | 13806 |
| Fugu | 10133 | 2549 | 576 | 13258 |
| Gilthead seabream | 10711 | 2870 | 495 | 14076 |
| Greater amberjack | 10750 | 2957 | 552 | 14259 |
| Guppy | 10623 | 2662 | 509 | 13794 |
| Huchen | 3797 | 6234 | 4510 | 14541 |
| Indian glassy fish | 10440 | 2833 | 556 | 13829 |
| Indian medaka | 10589 | 2798 | 531 | 13918 |
| Japanese medaka HdrR | 10699 | 2557 | 474 | 13730 |
| Japanese medaka HNI | 10528 | 2432 | 426 | 13386 |
| Japanese medaka HSOK | 10609 | 2563 | 455 | 13627 |
| Jewelled blenny | 9907 | 2882 | 881 | 13670 |
| Large yellow croaker | 10645 | 2916 | 464 | 14025 |
| Live sharksucker | 10656 | 2761 | 422 | 13839 |
| Lyretail cichlid | 10331 | 2816 | 640 | 13787 |
| Makobe Island cichlid | 10552 | 2881 | 537 | 13970 |
| Mexican tetra | 10464 | 3139 | 621 | 14224 |
| Midas cichlid | 10441 | 2761 | 595 | 13797 |
| Mummichog | 10489 | 2813 | 553 | 13855 |
| Nile tilapia | 10588 | 2803 | 574 | 13965 |
| Northern pike | 10447 | 3354 | 462 | 14263 |
| Orbiculate cardinalfish | 10081 | 2994 | 797 | 13872 |
| Pachon cavefish | 10333 | 2844 | 497 | 13674 |
| Pinecone soldierfish | 10486 | 3042 | 553 | 14081 |
| Rainbow trout | 4274 | 6304 | 3445 | 14023 |
| Red-bellied piranha | 10767 | 3201 | 589 | 14557 |
| Sailfin molly | 10238 | 2843 | 780 | 13861 |
| Sheepshead minnow | 10388 | 2800 | 597 | 13785 |

| | | | | |
|---|---|---|---|---|
| Shortfin molly | 10174 | 2962 | 780 | 13916 |
| Siamese fighting fish | 10663 | 2637 | 432 | 13732 |
| Stickleback | 10384 | 2474 | 337 | 13195 |
| Swamp eel | 10771 | 2672 | 512 | 13955 |
| Tetraodon | 9699 | 2576 | 643 | 12918 |
| Tiger tail seahorse | 10485 | 2519 | 512 | 13516 |
| Tongue sole | 10473 | 2615 | 555 | 13643 |
| Turbot | 10755 | 2757 | 340 | 13852 |
| Yellowtail amberjack | 10347 | 3084 | 735 | 14166 |
| Zebra mbuna | 10262 | 3037 | 831 | 14130 |
| Zebrafish | 10601 | 2942 | 552 | 14095 |
| Zig-zag eel | 10768 | 2867 | 431 | 14066 |
| **MAXIMAL** | 10868 | 7066 | 4772 | 14626 |
| **MINIMAL** | 3530 | 2323 | 337 | 12918 |
| **MOYENNE** | 9853,78947 | 3135,28070 | 892,82456 | 13881,89474 |

| X chromosome genes | | | |
|---|---|---|---|
| **Number of X chromosome teleost orthologs returned in singleton** | **Number of X chromosome teleost orthologs remained in duplicate** | **Number of X chromosome teleost orthologs remained in triplicate or more** | **Total number of X chromosome genes** |
| 122 | 30 | 4 | 156 |
| 112 | 35 | 3 | 150 |
| 37 | 79 | 46 | 162 |
| 116 | 34 | 8 | 158 |
| 110 | 40 | 6 | 156 |
| 126 | 32 | 2 | 160 |
| 123 | 25 | 4 | 152 |
| 39 | 78 | 45 | 162 |
| 129 | 26 | 4 | 159 |
| 120 | 28 | 3 | 151 |
| 132 | 31 | 2 | 165 |
| 124 | 31 | 3 | 158 |
| 120 | 28 | 2 | 150 |
| 38 | 71 | 50 | 159 |
| 113 | 35 | 11 | 159 |
| 123 | 27 | 5 | 155 |
| 123 | 34 | 2 | 159 |
| 122 | 30 | 0 | 152 |
| 125 | 25 | 4 | 154 |
| 121 | 32 | 0 | 153 |
| 126 | 29 | 2 | 157 |
| 125 | 29 | 2 | 156 |
| 32 | 78 | 54 | 164 |
| 122 | 33 | 3 | 158 |
| 127 | 31 | 3 | 161 |
| 131 | 26 | 1 | 158 |
| 126 | 26 | 1 | 153 |
| 127 | 25 | 2 | 154 |
| 123 | 28 | 5 | 156 |
| 127 | 32 | 2 | 161 |
| 129 | 25 | 3 | 157 |
| 126 | 29 | 3 | 158 |
| 124 | 30 | 3 | 157 |
| 114 | 44 | 3 | 161 |
| 124 | 30 | 2 | 156 |
| 127 | 23 | 5 | 155 |
| 124 | 29 | 3 | 156 |
| 114 | 43 | 2 | 159 |
| 122 | 29 | 6 | 157 |
| 111 | 42 | 2 | 155 |
| 126 | 29 | 4 | 159 |
| 42 | 78 | 39 | 159 |
| 118 | 42 | 4 | 164 |
| 126 | 32 | 3 | 161 |
| 132 | 24 | 4 | 160 |

| | | | |
|---|---|---|---|
| 127 | 29 | 4 | 160 |
| 125 | 27 | 3 | 155 |
| 121 | 26 | 1 | 148 |
| 128 | 26 | 5 | 159 |
| 110 | 33 | 1 | 144 |
| 121 | 32 | 2 | 155 |
| 125 | 25 | 5 | 155 |
| 128 | 29 | 2 | 159 |
| 124 | 30 | 5 | 159 |
| 121 | 33 | 6 | 160 |
| 122 | 38 | 2 | 162 |
| 132 | 25 | 6 | 163 |
| 132 | 79 | 54 | 165 |
| 32 | 23 | 0 | 144 |
| 115,50877 | 34,56140 | 7,14035 | 157,21053 |

**Comparison between singleton, duplicate, triplicate and more copies**

| Chi2 test | Chi2 test p-value | Chi2 test FDR (Benjamini-Hochberg procedure) | Hypergeometric test p-value | Hypergeometric test FDR (Benjamini-Hochberg procedure) |
|---|---|---|---|---|
| 1,4252441964 | 0,2325421590 | 0,5358397208 | 0,9037804847 | 0,9884503281 |
| 0,0007749713 | 0,9777911141 | 0,9777911141 | 0,5204207194 | 0,9884503281 |
| 1,4603177402 | 0,2268795717 | 0,5358397208 | 0,1291340065 | 0,9884503281 |
| 0,8162863499 | 0,3662681920 | 0,5358397208 | 0,8404096812 | 0,9884503281 |
| 0,1854944212 | 0,6666933307 | 0,7600303970 | 0,3614405237 | 0,9884503281 |
| 1,4287986592 | 0,2319605980 | 0,5358397208 | 0,9040324714 | 0,9884503281 |
| 1,5980960560 | 0,2061732362 | 0,5358397208 | 0,9169064225 | 0,9884503281 |
| 0,1976021253 | 0,6566633431 | 0,7600303970 | 0,3655547669 | 0,9884503281 |
| 2,1980089024 | 0,1381891322 | 0,5358397208 | 0,9465651825 | 0,9884503281 |
| 1,0053114091 | 0,3160287064 | 0,5358397208 | 0,8661727938 | 0,9884503281 |
| 0,8847556329 | 0,3469024498 | 0,5358397208 | 0,8510313397 | 0,9884503281 |
| 0,9425104321 | 0,3316330579 | 0,5358397208 | 0,8580368748 | 0,9884503281 |
| 0,0336166531 | 0,8545246232 | 0,9190170476 | 0,6055502713 | 0,9884503281 |
| 0,1453975440 | 0,7029730125 | 0,7856757199 | 0,3905705266 | 0,9884503281 |
| 0,0065192884 | 0,9356470467 | 0,9696705757 | 0,4978806621 | 0,9884503281 |
| 1,9087165206 | 0,1671058993 | 0,5358397208 | 0,9334918817 | 0,9884503281 |
| 0,4257974428 | 0,5140583172 | 0,6511405352 | 0,7708676140 | 0,9884503281 |
| 1,4644516350 | 0,2262231451 | 0,5358397208 | 0,9075792428 | 0,9884503281 |
| 1,9202366087 | 0,1658305791 | 0,5358397208 | 0,9347938758 | 0,9884503281 |
| 0,7523857034 | 0,3857219526 | 0,5358397208 | 0,8331480781 | 0,9884503281 |
| 2,0018735843 | 0,1571049089 | 0,5358397208 | 0,9380939788 | 0,9884503281 |
| 0,8558250237 | 0,3549096725 | 0,5358397208 | 0,8478939476 | 0,9884503281 |
| 3,7028569750 | 0,0543193836 | 0,5358397208 | 0,0297169721 | 0,9884503281 |
| 0,2531408592 | 0,6148721336 | 0,7456959918 | 0,7220193429 | 0,9884503281 |
| 0,6939524762 | 0,4048232419 | 0,5366261579 | 0,8235208834 | 0,9884503281 |
| 2,2843990657 | 0,1306807484 | 0,5358397208 | 0,9504959043 | 0,9884503281 |
| 1,2497827936 | 0,2635939666 | 0,5358397208 | 0,8914695859 | 0,9884503281 |
| 1,9020505876 | 0,1678489725 | 0,5358397208 | 0,9343962531 | 0,9884503281 |
| 3,1765300716 | 0,0747032133 | 0,5358397208 | 0,9727043867 | 0,9884503281 |
| 0,7825804364 | 0,3763531107 | 0,5358397208 | 0,8369927025 | 0,9884503281 |
| 2,3656858623 | 0,1240293912 | 0,5358397208 | 0,9530742764 | 0,9884503281 |
| 1,9493043731 | 0,1626618309 | 0,5358397208 | 0,9354847952 | 0,9884503281 |
| 1,0097663794 | 0,3149588138 | 0,5358397208 | 0,8660674339 | 0,9884503281 |
| 0,6299162345 | 0,4273860413 | 0,5536591898 | 0,2372721472 | 0,9884503281 |
| 1,2310564103 | 0,2672016898 | 0,5358397208 | 0,8884103467 | 0,9884503281 |
| 3,2709010954 | 0,0705188793 | 0,5358397208 | 0,9751548546 | 0,9884503281 |
| 1,1454384774 | 0,2845060759 | 0,5358397208 | 0,8804271051 | 0,9884503281 |
| 0,1942649292 | 0,6593904080 | 0,7600303970 | 0,3575766708 | 0,9884503281 |
| 2,0044483731 | 0,1568383378 | 0,5358397208 | 0,9374406379 | 0,9884503281 |
| 1,3123926789 | 0,2519619044 | 0,5358397208 | 0,1453017937 | 0,9884503281 |
| 1,9076974844 | 0,1672192503 | 0,5358397208 | 0,9332535673 | 0,9884503281 |
| 1,2389811038 | 0,2656675104 | 0,5358397208 | 0,1506171372 | 0,9884503281 |
| 0,3451643289 | 0,5568633341 | 0,6900263052 | 0,3044476769 | 0,9884503281 |
| 1,6137288039 | 0,2039684383 | 0,5358397208 | 0,9165026779 | 0,9884503281 |
| 4,3957624193 | 0,0360283473 | 0,5358397208 | 0,9884503281 | 0,9884503281 |

X Chromosome

| | | | | |
|---|---|---|---|---|
| 3,1943544406 | 0,0738929389 | 0,5358397208 | 0,9730642880 | 0,9884503281 |
| 0,8008441076 | 0,3708411150 | 0,5358397208 | 0,8406801930 | 0,9884503281 |
| 0,8267563513 | 0,3632121439 | 0,5358397208 | 0,8453029151 | 0,9884503281 |
| 0,9947892568 | 0,3185746487 | 0,5358397208 | 0,8648507067 | 0,9884503281 |
| 0,1316011127 | 0,7167776926 | 0,7856986246 | 0,6732391382 | 0,9884503281 |
| 0,0213743692 | 0,8837636968 | 0,9328616800 | 0,5898908577 | 0,9884503281 |
| 1,3085820579 | 0,2526515420 | 0,5358397208 | 0,8955877737 | 0,9884503281 |
| 0,7496939388 | 0,3865731488 | 0,5358397208 | 0,8329185148 | 0,9884503281 |
| 1,9755813287 | 0,1598566796 | 0,5358397208 | 0,9361037562 | 0,9884503281 |
| 0,7240174447 | 0,3948292679 | 0,5358397208 | 0,8273175633 | 0,9884503281 |
| 0,0008272669 | 0,9770542109 | 0,9777911141 | 0,5420031344 | 0,9884503281 |
| 1,7807311656 | 0,1820595691 | 0,5358397208 | 0,9271398266 | 0,9884503281 |
| 4,39576 | 0,97779 | 0,97779 | 0,98845 | 0,98845 |
| 0,00077 | 0,03603 | 0,53584 | 0,02972 | 0,98845 |
| 1,27767 | 0,35576 | 0,60195 | 0,74565 | 0,98845 |

| Comparison between tripli or more copies and dupli or less copies | |
|---|---|
| **Hypergeometric test p-value** | **Hypergeometric test FDR (Benjamini-Hochberg procedure)** |
| 0,9612438914 | 1,0000000000 |
| 0,9704698845 | 1,0000000000 |
| 0,5565964067 | 1,0000000000 |
| 0,9046347538 | 1,0000000000 |
| 0,9085976049 | 1,0000000000 |
| 0,9957344967 | 1,0000000000 |
| 0,8805491706 | 1,0000000000 |
| 0,3396552965 | 1,0000000000 |
| 0,8392150573 | 1,0000000000 |
| 0,9365777269 | 1,0000000000 |
| 0,9930292319 | 1,0000000000 |
| 0,9435340861 | 1,0000000000 |
| 0,9339243179 | 1,0000000000 |
| 0,7841156678 | 1,0000000000 |
| 0,3058024461 | 1,0000000000 |
| 0,9000371211 | 1,0000000000 |
| 0,9817342310 | 1,0000000000 |
| 1,0000000000 | 1,0000000000 |
| 0,9069220097 | 1,0000000000 |
| 1,0000000000 | 1,0000000000 |
| 0,9854854784 | 1,0000000000 |
| 0,9806772683 | 1,0000000000 |
| 0,3243935096 | 1,0000000000 |
| 0,9559402904 | 1,0000000000 |
| 0,9482372724 | 1,0000000000 |
| 0,9962408394 | 1,0000000000 |
| 0,9931056633 | 1,0000000000 |
| 0,9668394160 | 1,0000000000 |
| 0,9758491395 | 1,0000000000 |
| 0,9717047092 | 1,0000000000 |
| 0,8618592457 | 1,0000000000 |
| 0,9796519620 | 1,0000000000 |
| 0,9439139871 | 1,0000000000 |
| 0,9739740256 | 1,0000000000 |
| 0,9919581591 | 1,0000000000 |
| 0,7462599508 | 1,0000000000 |
| 0,9579323800 | 1,0000000000 |
| 0,9670040529 | 1,0000000000 |
| 0,8939194345 | 1,0000000000 |
| 0,9784645583 | 1,0000000000 |
| 0,8758019579 | 1,0000000000 |
| 0,5353203318 | 1,0000000000 |
| 0,9031756436 | 1,0000000000 |
| 0,9951429448 | 1,0000000000 |
| 0,9203217315 | 1,0000000000 |

| | |
|---|---|
| 0,9812899769 | 1,0000000000 |
| 0,8701178145 | 1,0000000000 |
| 0,9787371887 | 1,0000000000 |
| 0,6983732262 | 1,0000000000 |
| 0,9993852412 | 1,0000000000 |
| 0,9825771997 | 1,0000000000 |
| 0,7610721528 | 1,0000000000 |
| 0,9051386363 | 1,0000000000 |
| 0,9205656582 | 1,0000000000 |
| 0,9148378336 | 1,0000000000 |
| 0,9885644195 | 1,0000000000 |
| 0,3830100486 | 1,0000000000 |
| 1,00000 | 1,00000 |
| 0,30580 | 1,00000 |
| 0,87806 | 1,00000 |
