# Supplementary material for: Genes Encoding Teleost Orthologs of Human Haploinsufficient and Monoallelically Expressed Genes Remain in Duplicate More Frequently Than the Whole Genome
Source: Int J Genomics. 2021 Jul 29;2021:9028667. doi: 10.1155/2021/9028667 (PMC8346308; doi:10.1155/2021/9028667)
Supplement: Supplementary Materials — Tables of statistic tests for each species of teleost and for each category. Each category is represented in each of the four Suppl. Data (HI, MA, X chromosome, and parental imprinting), and these four tables have the same construction. By column (category HI for example): (A) species; (B) total number of teleost genes returned in singleton; (C) total number of teleost genes remained in duplicate; (D) total number of teleost genes in triplicate or more copies; (E) total number of teleost genes with a human ortholog; (F) number of teleost orthologs of HI human genes returned in singleton; (G) number of teleost orthologs of HI human genes remained in duplicate; (G) number of teleost orthologs of HI human genes in triplicate or more copies; (I) total number of teleost orthologs to human HI gene; (J) chi2 value of the repartition of HI orthologs in singleton, in duplicate or more copies in comparison with the whole genome; (K) p value of chi2 test; (L) chi2 false discovery rate (FDR) by Benjamini Hochberg (BH) procedure. (M) p value of hypergeometric test between singleton and duplicate/more copies; (N) hypergeometric FDR by BH procedure; (O) p value of hypergeometric test between triplicate or more copies and duplicate or less copies; (P) hypergeometric FDR by BH procedure. The same organization of columns is used for the other categories (MA, X chromosome, and imprinted genes). Concerning for HI and MA categories, the chi2 test is significant for 55/57 and 47/57 species, respectively, and the hypergeometric test is significant for 57/57 and 50/57 species, respectively; i.e., these orthologs remain more frequently in duplicate than the whole genome. For comparison between triplicate (or more copies) and duplicate (or less copies), the hypergeometric test is significant for 5/57 (salmonids and carp) and 3/57 species, respectively (salmonid and carp as well). [file 9028667.f1.zip › 9028667.f1/9028667.f3.pdf]

Species	Genome			
	Number of single copy genes	Number of duplicate copy genes	Number of triplicate or more copy genes	Total number of genes
Amazon molly	10313	2909	712	13934
Atlantic herring	10198	2818	624	13640
Atlantic salmon	3919	6418	4147	14484
Ballan wrasse	9759	3130	1027	13916
Barramundi perch	10123	3089	836	14048
Blue tilapia	10480	2910	651	14041
Blunt-snouted clingfish	10139	2552	549	13240
Brown trout	3744	7066	3816	14626
Burton's mouthbrooder	10678	2836	514	14028
Channel bull blenny	10109	2678	517	13304
Channel catfish	10868	2672	590	14130
Climbing perch	10638	2979	540	14157
Cod	10411	2323	379	13113
Common carp	3530	5699	4772	14001
Denticle herring	9916	3182	798	13896
Eastern happy	10464	2865	713	14042
Electric eel	10526	2973	513	14012
European seabass	10503	2861	442	13806
Fugu	10133	2549	576	13258
Gilthead seabream	10711	2870	495	14076
Greater amberjack	10750	2957	552	14259
Guppy	10623	2662	509	13794
Huchen	3797	6234	4510	14541
Indian glassy fish	10440	2833	556	13829
Indian medaka	10589	2798	531	13918
Japanese medaka HdrR	10699	2557	474	13730
Japanese medaka HNI	10528	2432	426	13386
Japanese medaka HSOK	10609	2563	455	13627
Jewelled blenny	9907	2882	881	13670
Large yellow croaker	10645	2916	464	14025
Live sharksucker	10656	2761	422	13839
Lyretail cichlid	10331	2816	640	13787
Makobe Island cichlid	10552	2881	537	13970
Mexican tetra	10464	3139	621	14224
Midas cichlid	10441	2761	595	13797
Mummichog	10489	2813	553	13855
Nile tilapia	10588	2803	574	13965
Northern pike	10447	3354	462	14263
Orbiculate cardinalfish	10081	2994	797	13872
Pachon cavefish	10333	2844	497	13674
Pinecone soldierfish	10486	3042	553	14081
Rainbow trout	4274	6304	3445	14023
Red-bellied piranha	10767	3201	589	14557
Sailfin molly	10238	2843	780	13861
Sheepshead minnow	10388	2800	597	13785

Shortfin molly	10174	2962	780	13916
Siamese fighting fish	10663	2637	432	13732
Stickleback	10384	2474	337	13195
Swamp eel	10771	2672	512	13955
Tetraodon	9699	2576	643	12918
Tiger tail seahorse	10485	2519	512	13516
Tongue sole	10473	2615	555	13643
Turbot	10755	2757	340	13852
Yellowtail amberjack	10347	3084	735	14166
Zebra mbuna	10262	3037	831	14130
Zebrafish	10601	2942	552	14095
Zig-zag eel	10768	2867	431	14066
[redacted]				
**MAXIMAL**	10868	7066	4772	14626
**MINIMAL**	3530	2323	337	12918
**MOYENNE**	9853,78947	3135,28070	892,82456	13881,89474

Parental imprinting genes			
**Number of parental imprinting teleost orthologs returned in singleton**	**Number of parental imprinting teleost orthologs remained in duplicate**	**Number of parental imprinting teleost orthologs remained in triplicate or more**	**Total number of parental imprinting genes**
40	7	1	48
36	5	2	43
15	20	12	47
35	7	3	45
40	5	3	48
38	8	1	47
33	7	2	42
14	22	11	47
37	9	1	47
39	8	0	47
35	10	3	48
40	7	0	47
35	6	0	41
12	23	14	49
34	10	4	48
40	7	0	47
36	6	2	44
37	5	3	45
31	11	2	44
38	7	1	46
35	9	1	45
36	7	3	46
12	15	20	47
40	5	3	48
38	5	4	47
38	3	5	46
39	3	3	45
37	4	4	45
35	6	4	45
40	7	0	47
36	6	2	44
38	6	2	46
37	8	2	47
37	6	3	46
36	7	2	45
38	8	1	47
36	10	0	46
37	8	1	46
36	8	3	47
34	7	2	43
41	5	2	48
16	19	10	45
38	9	1	48
37	8	1	46
39	8	0	47

39	8	1	48
38	7	1	46
34	6	0	40
36	6	1	43
32	5	2	39
38	7	2	47
35	7	2	44
37	7	0	44
38	7	2	47
39	8	0	47
38	8	0	46
38	7	1	46
[redacted]			
41	23	20	49
12	3	0	39
34,96491	8,07018	2,73684	45,77193

Comparison between singleton, duplicate, triplicate and more copies

Chi2 test	Chi2 test p-value	Chi2 test FDR (Benjamini-Hochberg procedure)	Hypergeometric test p-value	Hypergeometric test FDR (Benjamini-Hochberg procedure)
2,16781931652	0,14092594834	0,64434095476	0,9552571332	0,9775683169
1,82790798273	0,17637484410	0,64434095476	0,9430909017	0,9775683169
0,56188439137	0,45350198755	0,65460543338	0,8211496266	0,9775683169
1,25708265097	0,26220402818	0,64434095476	0,9039127911	0,9775683169
3,02984136732	0,08174600839	0,64434095476	0,9766520190	0,9775683169
0,95828398003	0,32761960679	0,64434095476	0,8769631780	0,9775683169
0,09300153399	0,76039545311	0,81778378919	0,6779087930	0,9775683169
0,43303391380	0,51050399575	0,66197116796	0,7982018729	0,9775683169
0,17535090386	0,67539942470	0,76995534416	0,7157423413	0,9775683169
1,25991609315	0,26166697361	0,64434095476	0,9068013864	0,9775683169
0,43202846256	0,51099528755	0,66197116796	0,3051302264	0,9775683169
2,49787865671	0,11399976162	0,64434095476	0,9664453344	0,9775683169
0,89362774798	0,34449611238	0,64434095476	0,8761637799	0,9775683169
0,01357232029	0,90725604703	0,93583402318	0,5299965188	0,9775683169
0,00648137825	0,93583402318	0,93583402318	0,5224688781	0,9775683169
2,77440861278	0,09578202804	0,64434095476	0,9728418984	0,9775683169
1,05585496674	0,30416247840	0,64434095476	0,8889297788	0,9775683169
0,93410700615	0,33379799117	0,64434095476	0,8760501929	0,9775683169
0,87189565398	0,35043104557	0,64434095476	0,2204489022	0,9775683169
1,07320185620	0,30022334069	0,64434095476	0,8904645906	0,9775683169
0,13817505816	0,71010203388	0,77838107560	0,6997915280	0,9775683169
0,04054215977	0,84042425385	0,88711449018	0,6364183444	0,9775683169
0,00820767112	0,92781345293	0,93583402318	0,5405664371	0,9775683169
1,59463492247	0,20666518063	0,64434095476	0,9294644628	0,9775683169
0,58758462806	0,44335465091	0,65460543338	0,8253785111	0,9775683169
0,58679384563	0,44366160367	0,65460543338	0,8270443176	0,9775683169
1,72252582204	0,18936824063	0,64434095476	0,9399710719	0,9775683169
0,49827478158	0,48025915058	0,66197116796	0,8105351216	0,9775683169
0,63486054702	0,42557801838	0,65460543338	0,8324675544	0,9775683169
2,17772225096	0,14002155581	0,64434095476	0,9569529489	0,9775683169
0,57681519451	0,44756337761	0,65460543338	0,8256018817	0,9775683169
1,44287002784	0,22967547166	0,64434095476	0,9201187661	0,9775683169
0,25881980135	0,61093212875	0,72548190289	0,7463421428	0,9775683169
1,11609210523	0,29076148354	0,64434095476	0,8929666713	0,9775683169
0,45710393917	0,49898057037	0,66197116796	0,8003237380	0,9775683169
0,67658916138	0,41076371424	0,65460543338	0,8401159253	0,9775683169
0,14971102162	0,69881166945	0,77838107560	0,7048683344	0,9775683169
1,21326465503	0,27068652780	0,64434095476	0,9015110232	0,9775683169
0,36443122889	0,54605548469	0,69167028061	0,7757330661	0,9775683169
0,28578312270	0,59293556417	0,71909206718	0,7571928030	0,9775683169
3,02573776814	0,08195303977	0,64434095476	0,9775683169	0,9775683169
0,54742382152	0,45937223395	0,65460543338	0,8178773850	0,9775683169
0,67458032455	0,41145924068	0,65460543338	0,8382927611	0,9775683169
1,02937426257	0,31030568071	0,64434095476	0,8845732874	0,9775683169
1,47014183203	0,22532331714	0,64434095476	0,9218802658	0,9775683169

1,61775914166	0,20340452658	0,64434095476	0,9293912446	0,9775683169
0,65155924326	0,41955576048	0,65460543338	0,8379398246	0,9775683169
0,94802564449	0,33022236217	0,64434095476	0,8823911840	0,9775683169
1,04345241940	0,30701988770	0,64434095476	0,8896693332	0,9775683169
1,01268132802	0,31426133166	0,64434095476	0,8869704490	0,9775683169
0,29001278198	0,59021233940	0,71909206718	0,7574096448	0,9775683169
0,19075931073	0,66228539982	0,76995534416	0,7241481690	0,9775683169
1,05406939974	0,30457172561	0,64434095476	0,8906432257	0,9775683169
1,45588817316	0,22758549021	0,64434095476	0,9194995633	0,9775683169
2,53398985696	0,11141816997	0,64434095476	0,9661604630	0,9775683169
1,35021591209	0,24524037432	0,64434095476	0,9136819094	0,9775683169
0,93969157572	0,33235717311	0,64434095476	0,8765769657	0,9775683169
[redacted]				
3,02984	0,93583	0,93583	0,97757	0,97757
0,00648	0,08175	0,64434	0,22045	0,97757
0,99444	0,39979	0,68383	0,81934	0,97757

Comparison between tripli or more copies and dupli or less copies	
Hypergeometric test p-value	Hypergeometric test FDR (Benjamini-Hochberg procedure)
0,91969788372	1,00000000000
0,59167155691	1,00000000000
0,73161853524	1,00000000000
0,65528084479	1,00000000000
0,55040331717	1,00000000000
0,89300832430	1,00000000000
0,52468586208	1,00000000000
0,71496448977	1,00000000000
0,82750852911	1,00000000000
1,00000000000	1,00000000000
0,32443885186	1,00000000000
1,00000000000	1,00000000000
1,00000000000	1,00000000000
0,83283033066	1,00000000000
0,29658562524	1,00000000000
1,00000000000	1,00000000000
0,48254549653	1,00000000000
0,17389424576	1,00000000000
0,57567927026	1,00000000000
0,80784657953	1,00000000000
0,83127338931	1,00000000000
0,24038774061	1,00000000000
0,06267636237	1,00000000000
0,30386035497	1,00000000000
0,10347346240	1,00000000000
0,02056074564	1,00000000000
0,17176571598	1,00000000000
0,06243174860	1,00000000000
0,32950431769	1,00000000000
1,00000000000	1,00000000000
0,38990662355	1,00000000000
0,63666567736	1,00000000000
0,54422489405	1,00000000000
0,32555884824	1,00000000000
0,58388595259	1,00000000000
0,85304731505	1,00000000000
1,00000000000	1,00000000000
0,78065500981	1,00000000000
0,51181036217	1,00000000000
0,46668689685	1,00000000000
0,56749771847	1,00000000000
0,69752313276	1,00000000000
0,86273168483	1,00000000000
0,93065813259	1,00000000000
1,00000000000	1,00000000000

0,93756281335	1,00000000000
0,77072573312	1,00000000000
1,00000000000	1,00000000000
0,80006867679	1,00000000000
0,58493909878	1,00000000000
0,53622892310	1,00000000000
0,53943407033	1,00000000000
1,00000000000	1,00000000000
0,70849660857	1,00000000000
1,00000000000	1,00000000000
1,00000000000	1,00000000000
0,76161660669	1,00000000000
[redacted]	
1,00000	1,00000
0,02056	1,00000
0,64594	1,00000
