# Supplementary material for: Genes Encoding Teleost Orthologs of Human Haploinsufficient and Monoallelically Expressed Genes Remain in Duplicate More Frequently Than the Whole Genome
Source: Int J Genomics. 2021 Jul 29;2021:9028667. doi: 10.1155/2021/9028667 (PMC8346308; doi:10.1155/2021/9028667)
Supplement: Supplementary Materials — Tables of statistic tests for each species of teleost and for each category. Each category is represented in each of the four Suppl. Data (HI, MA, X chromosome, and parental imprinting), and these four tables have the same construction. By column (category HI for example): (A) species; (B) total number of teleost genes returned in singleton; (C) total number of teleost genes remained in duplicate; (D) total number of teleost genes in triplicate or more copies; (E) total number of teleost genes with a human ortholog; (F) number of teleost orthologs of HI human genes returned in singleton; (G) number of teleost orthologs of HI human genes remained in duplicate; (G) number of teleost orthologs of HI human genes in triplicate or more copies; (I) total number of teleost orthologs to human HI gene; (J) chi2 value of the repartition of HI orthologs in singleton, in duplicate or more copies in comparison with the whole genome; (K) p value of chi2 test; (L) chi2 false discovery rate (FDR) by Benjamini Hochberg (BH) procedure. (M) p value of hypergeometric test between singleton and duplicate/more copies; (N) hypergeometric FDR by BH procedure; (O) p value of hypergeometric test between triplicate or more copies and duplicate or less copies; (P) hypergeometric FDR by BH procedure. The same organization of columns is used for the other categories (MA, X chromosome, and imprinted genes). Concerning for HI and MA categories, the chi2 test is significant for 55/57 and 47/57 species, respectively, and the hypergeometric test is significant for 57/57 and 50/57 species, respectively; i.e., these orthologs remain more frequently in duplicate than the whole genome. For comparison between triplicate (or more copies) and duplicate (or less copies), the hypergeometric test is significant for 5/57 (salmonids and carp) and 3/57 species, respectively (salmonid and carp as well). [file 9028667.f1.zip › 9028667.f1/9028667.f4.pdf]

Species	Genome				Number of monoallelic teleost orthologs returned in singleton
	Number of single copy genes	Number of duplicate copy genes	Number of triplicate or more copy genes	Total number of genes	
Amazon molly	10313	2909	712	13934	271
Atlantic herring	10198	2818	624	13640	285
Atlantic salmon	3919	6418	4147	14484	105
Ballan wrasse	9759	3130	1027	13916	270
Barramundi perch	10123	3089	836	14048	279
Blue tilapia	10480	2910	651	14041	275
Blunt-snouted clingfish	10139	2552	549	13240	272
Brown trout	3744	7066	3816	14626	101
Burton's mouthbrooder	10678	2836	514	14028	283
Channel bull blenny	10109	2678	517	13304	272
Channel catfish	10868	2672	590	14130	296
Climbing perch	10638	2979	540	14157	287
Cod	10411	2323	379	13113	288
Common carp	3530	5699	4772	14001	87
Denticle herring	9916	3182	798	13896	265
Eastern happy	10464	2865	713	14042	282
Electric eel	10526	2973	513	14012	290
European seabass	10503	2861	442	13806	286
Fugu	10133	2549	576	13258	262
Gilthead seabream	10711	2870	495	14076	278
Greater amberjack	10750	2957	552	14259	285
Guppy	10623	2662	509	13794	289
Huchen	3797	6234	4510	14541	102
Indian glassy fish	10440	2833	556	13829	276
Indian medaka	10589	2798	531	13918	279
Japanese medaka HdrR	10699	2557	474	13730	293
Japanese medaka HNI	10528	2432	426	13386	292
Japanese medaka HSOK	10609	2563	455	13627	286
Jewelled blenny	9907	2882	881	13670	268
Large yellow croaker	10645	2916	464	14025	288
Live sharksucker	10656	2761	422	13839	284
Lyretail cichlid	10331	2816	640	13787	282
Makobe Island cichlid	10552	2881	537	13970	290
Mexican tetra	10464	3139	621	14224	280
Midas cichlid	10441	2761	595	13797	281
Mummichog	10489	2813	553	13855	284
Nile tilapia	10588	2803	574	13965	277
Northern pike	10447	3354	462	14263	272
Orbiculate cardinalfish	10081	2994	797	13872	271
Pachon cavefish	10333	2844	497	13674	296
Pinecone soldierfish	10486	3042	553	14081	288
Rainbow trout	4274	6304	3445	14023	115
Red-bellied piranha	10767	3201	589	14557	288
Sailfin molly	10238	2843	780	13861	266
Sheepshead minnow	10388	2800	597	13785	286
Shortfin molly	10174	2962	780	13916	270

Siamese fighting fish	10663	2637	432	13732	281
Stickleback	10384	2474	337	13195	289
Swamp eel	10771	2672	512	13955	279
Tetraodon	9699	2576	643	12918	270
Tiger tail seahorse	10485	2519	512	13516	285
Tongue sole	10473	2615	555	13643	268
Turbot	10755	2757	340	13852	290
Yellowtail amberjack	10347	3084	735	14166	268
Zebra mbuna	10262	3037	831	14130	281
Zebrafish	10601	2942	552	14095	287
Zig-zag eel	10768	2867	431	14066	280
**MAXIMAL**	10868	7066	4772	14626	296
**MINIMAL**	3530	2323	337	12918	87
**MEAN**	9853.78947	3135.2807	892.82456	13881.89474	264.91228

Monoallelic genes			
Number of monoallelic teleost orthologs remained in duplicate	Number of monoallelic teleost orthologs remained in triplicate or more	Total number of monoalleleic genes	Chi2 test
121	19	411	13.9387096260202
98	11	394	1.23351394573779
188	139	432	1.65759050976307
103	37	410	3.57558871003738
112	25	416	5.15058909271909
125	9	409	11.8362262777822
109	13	394	12.4987176965819
193	136	430	1.00505436615535
117	9	409	10.7931846664825
105	17	394	10.4266048912999
93	18	407	4.01856077675943
109	19	415	7.96241030696846
87	4	379	2.68603733183254
163	160	410	3.46682379717688
115	22	402	5.81710660555946
110	19	411	7.55043877371843
114	16	420	8.29022397650593
119	10	415	11.6891432474081
113	16	391	19.2663274731695
118	15	411	16.1482693430771
119	13	417	11.1574616489647
101	15	405	7.31241053606669
174	154	430	1.27458850248621
110	17	403	10.6954866714656
119	10	408	13.289588471646
104	11	408	8.85583642810311
98	10	400	7.60235610699769
105	11	402	10.492657449668
115	26	409	9.89385319704292
109	14	411	7.62961968920492
112	10	406	11.3910847293066
108	20	410	8.26226773647726
109	12	411	5.50151857284609
130	7	417	8.83688604845189
112	13	406	9.21596000684082
115	15	414	11.3677683829423
115	17	409	14.6072058798889
130	9	411	10.4698042534466
120	23	414	10.8444762562646
102	9	407	1.77731856252715
114	12	414	5.23656265697537
187	118	420	1.90183328264716
126	10	424	8.0321791846593
118	19	403	12.8860231673662
113	14	413	8.29691752606091
115	23	408	9.97730787470275

106	11	398	11.3912818606198
101	7	397	8.24446175422885
121	6	406	16.5184319794668
92	18	380	3.29644150913356
100	10	395	6.67714292991364
113	13	394	16.8903773302179
107	6	403	7.49494785386498
126	22	416	15.6905229960926
113	22	416	5.39468739044395
108	17	412	6.80895265218264
122	8	410	15.5874097052721
193	160	432	19.26633
87	4	379	1.00505
117.5614	25.7193	408.19298	8.76868

Comparison between singleton. duplicate. triplicate and more copies		
Chi2 test p-value	Chi2 test FDR (Benjamini-Hochberg procedure)	Hypergeometric test p-value
0.00018886860127149	0.00134568878405937	0.000137464383867894
0.266724746147075	0.271487688042558	0.142965724384076
0.197928962638283	0.208925016118188	0.104376117577757
0.0586346618682334	0.0696286609685272	0.0322284554379218
0.0232384880153939	0.0287955177582055	0.0131983979275941
0.000580894308096017	0.00266224236437687	0.000402737082218547
0.000407231436306382	0.00211019926086034	0.000301752058704081
0.316090582201079	0.316090582201079	0.168291973747208
0.00101874467130022	0.00305623401390067	0.000701654596899842
0.00124212871655956	0.00307831899321282	0.000847898227353685
0.0450021009164876	0.054577016005102	0.0257145725186945
0.00477587727939797	0.00800661779193189	0.00296943604770668
0.101231283926359	0.113140846741225	0.0568461637573481
0.0626114256052774	0.0728336991734859	0.0317707364061847
0.0158710321529334	0.0215392579218382	0.00914170841516215
0.00599954641218754	0.00924254447282945	0.00367156367903466
0.0039859072213287	0.0072811316082688	0.00248731259369759
0.000628658395851695	0.00266224236437687	0.000441640625874871
0.0000113693892700165	0.00064805518839094	0.0000110329451590078
0.0000585720637532463	0.00074838526850211	0.0000487343412091267
0.000836943600753705	0.00280622266135066	0.000573052662348618
0.00684800045720776	0.0100086160528421	0.00428266166196166
0.258907641673038	0.268322465006603	0.137396732418724
0.00107397217654475	0.00306082070315253	0.000731847894935271
0.000266884055951835	0.00169026568769495	0.000199967136840418
0.00292153211347105	0.0060094774957342	0.00193655674575803
0.00582920771639041	0.00922957888428482	0.00375053682402355
0.00119849868506232	0.00307831899321282	0.000844168768457975
0.00165831764169393	0.00378096422306216	0.00104757283893488
0.00574174085929846	0.00922957888428482	0.00357274007335961
0.000737974249417222	0.00266224236437687	0.000527605536828009
0.00404775337944835	0.0072811316082688	0.00253116744629472
0.0189999669939578	0.0251860027594324	0.0111355190117333
0.00295202403299224	0.0060094774957342	0.00184218830015479
0.00239914374388519	0.00525966128467137	0.00156148171712992
0.000747296102281226	0.00266224236437687	0.000516792581451189
0.000132407278526917	0.00107817355371918	0.000103296208374819
0.00121341575026333	0.00307831899321282	0.000790756986779675
0.000990909026231308	0.00305623401390067	0.000641556215148135
0.182478936531605	0.196250931741538	0.0986881217365519
0.0221169316129434	0.0280147800430616	0.0127804679216711
0.167873259864098	0.184014919466416	0.0881609735128426
0.00459535187644055	0.0079374259683973	0.00281853107038826
0.000331045112485734	0.00188695714116868	0.000234350502310824
0.00397124262792783	0.0072811316082688	0.00249262706919953
0.00158481227777903	0.0037639291597252	0.00101598320651583

0.000737895939414840	0.00266224236437687	0.000538208305466549
0.00408765283271231	0.0072811316082688	0.00269165066468724
0.0000481794330783668	0.00074838526850211	0.0000423005813864691
0.0694301408849763	0.0791503606088729	0.0388746981490364
0.00976570314494789	0.0135767092502934	0.00605247372300639
0.0000396018366304259	0.00074838526850211	0.0000352750946425042
0.00618723228566243	0.00928084842849364	0.00392687051045541
0.0000745970034529486	0.00074838526850211	0.0000558451630491113
0.0201981432735984	0.0261657765135251	0.0115694085840062
0.00907019493624454	0.0129250277841485	0.00548144181437892
0.0000787773966844327	0.00074838526850211	0.0000650920324267127
0.31609	0.31609	0.16829
0.00001	0.00065	0.00001
0.0338	0.0378	0.01835

	Comparison between tripli or more copies and dupli or less copies	
**Hypergeometric test FDR (Benjamini-Hochberg procedure)**	**Hypergeometric test p-value**	**Hypergeometric test FDR (Benjamini-Hochberg procedure)**
0.000979433735058742	0.707690934057916	0.999344539373478
0.145518683748077	0.974308363306199	0.999344539373478
0.110174790776522	0.0559093356118618	0.637366425975224
0.0374902440808478	0.117459032564569	0.999344539373478
0.0163545365624536	0.509863318505342	0.999344539373478
0.0019130011405381	0.99695791187862	0.999344539373478
0.00156362430419387	0.8377279944677	0.999344539373478
0.168291973747208	0.00530186120487802	0.302206088678047
0.00208576650056552	0.966685262815658	0.999344539373478
0.00210131299822435	0.363559405920082	0.999344539373478
0.03118575816097	0.435330527411394	0.999344539373478
0.00497817219762591	0.237484152169085	0.999344539373478
0.0635339477288008	0.995745472692328	0.999344539373478
0.0374902440808478	0.0190592413693036	0.362125586016768
0.0124066042777201	0.624673418135437	0.999344539373478
0.00577785402619844	0.697414975305867	0.999344539373478
0.00465408207867094	0.471696523553936	0.999344539373478
0.00191736708822458	0.860357779453336	0.999344539373478
0.000618374308053771	0.63410987881896	0.999344539373478
0.000618374308053771	0.479007577066066	0.999344539373478
0.00192141186787478	0.8252622825571	0.999344539373478
0.00625927473671319	0.531904793268275	0.999344539373478
0.14239297723395	0.0174090714300202	0.362125586016768
0.00208576650056552	0.454787619238195	0.999344539373478
0.00126645853332265	0.952136512110961	0.999344539373478
0.003942276232436	0.838477843537542	0.999344539373478
0.00577785402619844	0.82391402588158	0.999344539373478
0.00210131299822435	0.791289079546491	0.999344539373478
0.00238846607277153	0.558941883455945	0.999344539373478
0.00577785402619844	0.494359357741465	0.999344539373478
0.00191736708822458	0.797356726625767	0.999344539373478
0.00465408207867094	0.44237460483168	0.999344539373478
0.014761036829507	0.871351973991047	0.999344539373478
0.00388906418921566	0.999344539373478	0.999344539373478
0.00342324837986175	0.89737804911847	0.999344539373478
0.00191736708822458	0.687328925530021	0.999344539373478
0.000841126268194953	0.51676795381586	0.999344539373478
0.00210131299822435	0.920267103680643	0.999344539373478
0.00203159468130243	0.59785511013618	0.999344539373478
0.106136281867612	0.963040067896808	0.999344539373478
0.0161885927007834	0.893610356551591	0.999344539373478
0.0966379901967698	0.0511815632996389	0.637366425975224
0.00486837184885244	0.979356294324824	0.999344539373478
0.0013357978631717	0.819238455982393	0.999344539373478
0.00465408207867094	0.861010885366658	0.999344539373478
0.00238846607277153	0.519950431694409	0.999344539373478

0.00191736708822458	0.712287896855226	0.999344539373478
0.00479450274647415	0.885280843359738	0.999344539373478
0.000618374308053771	0.99762551886754	0.999344539373478
0.0443171558899014	0.621014182525859	0.999344539373478
0.00841441468808205	0.935467435349897	0.999344539373478
0.000618374308053771	0.818050594333129	0.999344539373478
0.00589030576568311	0.933906601806451	0.999344539373478
0.000618374308053771	0.494861680954577	0.999344539373478
0.0149876429383716	0.728813668838194	0.999344539373478
0.00781105458548996	0.447833086770088	0.999344539373478
0.000618374308053771	0.938017521986449	0.999344539373478
0.16829	0.99934	0.99934
0.00062	0.0053	0.30221
0.02074	0.65981	0.95205
